# Prediction of Whole Liver Graft Weight Based on Biometric Variables in Paediatric and Adult Liver Donors

**DOI:** 10.3390/children11101248

**Published:** 2024-10-16

**Authors:** Maria Kuksin, Valeska Bidault Jourdainne, Guillaume Rossignol, Philippe Aegerter, Géraldine Hery, Jean-Paul Teglas, Virginie Fouquet, Sophie Branchereau, Florent Guérin

**Affiliations:** 1 Department of Clinical Research, Assistance Publique—Hôpitaux de Paris, Université Paris-Saclay, Bicêtre Hospital, 78 Rue du Général Leclerc, 94270 Le Kremlin Bicêtre, France; maria.kuksin@ens-lyon.fr (M.K.); jean-paul.teglas@inserm.fr (J.-P.T.); 2 Department of Paediatric Surgery, Hôpital Mère-Enfant, Hospices Civils de Lyon, 59 Boulevard Pinel, 69500 Bron, France; valeska.bidault@chu-lyon.fr (V.B.J.); guillaume.rossignol@chu-lyon.fr (G.R.); 3 Department of Public Health—U1018 UVSSQ INSERM, GIRCI IdF—UFR Médecine Paris—Ile de France Ouest Université Versailles Saint Quentin, 9 Avenue Charles de Gaulle, 92100 Boulogne, France; philippe.aegerter@uvsq.fr; 4 Department of Paediatric Surgery, Assistance Publique—Hôpitaux de Paris, Université Paris-Saclay, Bicêtre Hospital, 78 Rue du Général Leclerc, 94270 Le Kremlin Bicêtre, France; geraldine.hery@aphp.fr (G.H.); virginie.fouquet@aphp.fr (V.F.); sophie.branchereau@aphp.fr (S.B.)

**Keywords:** liver transplantation, model, linear regression

## Abstract

Background/Objectives: In paediatric liver transplantation, donor–recipient compatibility depends on graft size. We explored whether the graft weight can be predicted using the donor’s biometric parameters. Methods: We used seven easily available biometric variables in 142 anonymised paediatric and adult donors, with data collected between 2016 and 2022. The whole or partial liver was transplanted in our hospital from these donors. We identified the variables that had the strongest correlation to our response variable: whole liver graft weight. Results: In child donors, we determined two linear models: using donor weight and height on the one hand and using donor weight and right liver span on the other hand. Both models had a coefficient of determination R^2^ = 0.86 and *p*-value < 10^−5^. We also determined two models in adult donors using donor weight and height (R^2^ = 0.33, *p* < 10^−4^) and donor weight and sternal height (R^2^ = 0.38, *p* < 10^−4^). The models proved valid based on our external dataset of 245 patients from two institutions. Conclusions: In clinical practise, our models could provide rapidly accessible estimates to determine whole graft dimension compatibility in liver transplantation in children and adults. Determining similar models predicting the left lobe and lateral segment weight could prove invaluable in paediatric transplantation.

## 1. Introduction

End-stage liver disease is a common cause of morbidity and mortality, making up 2% of total annual deaths worldwide in 2010 [1]. It is, to date, incurable using medical treatments. With survival rates of, respectively, 95% and 85% at one and five years, liver transplantation has become the key treatment strategy [2].

As in all organ transplantations, donor–recipient compatibility depends on molecular and genetic parameters. A specificity of the liver resides in its inter-individual diversity in terms of size. In the recipient, while an undersized liver can result in small-for-size syndrome, an excessively large liver can cause large-for-size syndrome [3,4,5]. In both cases, the graft dysfunctions, resulting in clinical symptoms of acute liver failure such as cholestasis, coagulopathy, portal hypertension, and ascites [6,7]. Predicting liver size prior to transplantation is essential to the operation’s success, especially in paediatric liver transplantation, where the recipient’s age influences the liver weight.

Liver volume can be calculated using Computer Tomography (CT) scan imaging. The process is tedious and consists of delineating an area of interest on CT scan slides. It has warranted the development of machine learning algorithms attempting to optimise the job [3]. Both human and machine-learning-based determinations are prone to errors. CT estimation tends to over- or underestimate the liver volume depending on the phase of contrast enhancement, individual properties, abdominal organ densities, and CT section thickness [8]. Moreover, such time-consuming calculations are not adapted to the urgent decision-making process of accepting a graft for transplantation.

The technical challenges and unreliability of these techniques call for developing other solutions. Some authors have suggested regressions to estimate liver volumes using other donor parameters [4,9,10]. An accurate mathematical model to estimate liver weight or volume would prove invaluable. It could offer a widely accessible, rapidly applicable, and cheap solution to perform better-suited liver transplantations. Once it is built, a mathematical model can be made to evolve as larger numbers of donors are included, constantly improving its proficiency in the modelled population. To our knowledge, no model granting clinically sufficient precision and reliability in modelling liver graft weights in the Caucasian population has been established yet.

The goal of our study was to develop a mathematical model predicting whole liver weights in both paediatric and adult donors, using only a few widely accessible biometrics variables, through our national harvesting system.

## 2. Materials and Methods

From 2016 to 2020, we recorded the biometric data of the donors accepted for liver transplantation in our team, which is the largest paediatric liver transplantation centre in our country. Since 2016, our national allocation system has required donors to undergo routine CT scans. These scans are performed at the local hospital, where the on-call radiologist measures and records basic parameters such as the right hepatic height, liver–spleen density gradient, and variation in the vascularisation of the liver. However, it was only possible to view the imaging starting in 2018 using a basic image viewer that does not allow for reconstruction, meaning that the volumetric tool is not available. Apart from acute liver failure, in the context of paediatric liver transplantation, particularly when splitting the liver and increasing the ischemia time, our team has a practise of rejecting adult steatotic livers based on CT scan imaging or a BMI above 30 associated with abnormal liver function tests. The outcome variable was the whole liver graft weight, measured using the same scales for each graft. Each graft consisted of the whole liver with the coeliac trunk after the removal of attached organs if harvested with the liver (diaphragm, pancreas…). The training dataset consisted of 44 paediatric donors (≤18 years old) and 98 adult donors. Two individuals were excluded from analysis, as their liver weight over 2000 g and donor age over 60 years were above the 99.5th percentile of the donor population. Analyses were performed using RStudio Version 2023.06.1+524, and the scripts can be made available upon reasonable demand. Departures from linearity were explored by fractional polynomials and smoothing functions. The external validation dataset contained 101 paediatric and 144 adult donors from our institution and third largest paediatric liver transplantation teams in our country. The available donor variables (systematically reported by our national harvesting system during the decision process for graft acceptation) were age (years), sex, height (cm), donor weight (kg), length of right liver span (cm) (vertical length of liver measured on a coronal CT scan plan, along the midclavicular line), chest perimeter (cm) (measured at nipple level), sternal height (cm), and abdominal perimeter (cm) (measured at navel level).

## 3. Results

### 3.1. Patient Characteristics

A total of 142 donors were included in this study’s training dataset. Table 1 and Table 2 report their principal characteristics.

In the training dataset, the median age was 12 years in the under-eighteen population (range 0 to 18) and 32 years in the over-eighteen population (range 19 to 59). In the paediatric group, there were 31 males (70%). In the adult population, there were 45 males (47%).

A further 245 donors were included in the external validation dataset (Table 3 and Table 4).

In the paediatric population, the median age was 12 years (range 0 to 18), and in the adult population, the median age was 29 (range 19 to 73). There were 66 males in the paediatric population (65%) and 73 among the adults (51%).

### 3.2. Training of Whole Graft Model

**Figure 1:**
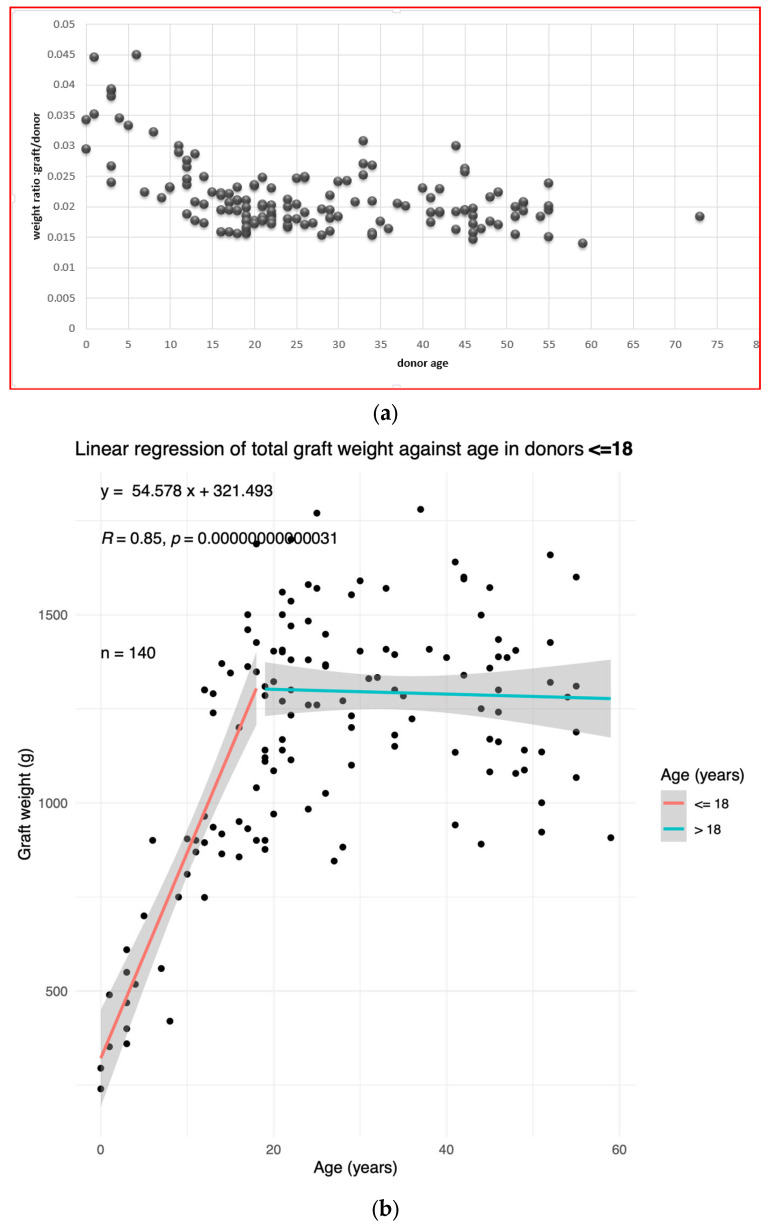
The liver weight depends on the donor age. (**a**) The ratio of liver graft weight to donor weight as a function of the donor age in the training population. (**b**) The graft weight plotted against the donor age in the training population. The linear regression equation, correlation coefficient, and *p*-value in donors ≤ 18 are shown in top left corner.

First, we performed an overview of how the graft weight over donor weight ratio evolves with age (Figure 1a). The ratio decreases in childhood. In adulthood, the graft weight/donor weight ratio seems stable, with inter-individual variations.

Next, the liver weight was plotted against age. The resulting plot is that of a piecewise continuous function. We drew the cut-off at 18 y. In individuals aged ≤ 18, the liver weight is strongly correlated to age (Pearson correlation coefficient R = 0.85, *p*-value < 10^−5^) (Figure 1b).

In adults > 18, the liver weight does not vary significantly with age. We therefore separated the population into ≤18 and >18 yo age groups.

**Figure 2:**
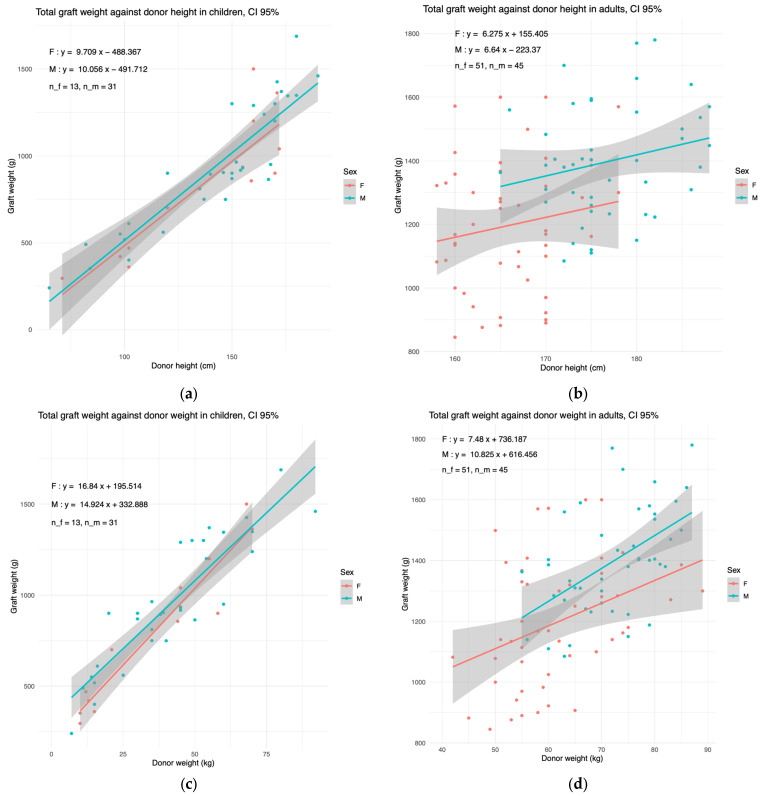
The graft weight evolves similarly with donor weight and donor height in females and males. The linear regressions of total graft weight in females (F) and males (M), plotted against (**a**,**b**) the donor height and (**c**,**d**) donor weight in (**a**,**c**) children and (**b**,**d**) in adults. The 95% confidence interval is shown in grey. The number of plotted females (n_f) and males (n_m) are shown in top left.

We next explored whether the liver weight should be stratified by sex. In children, the donor weight and height are variables that vary strongly with age. We therefore plotted the liver weight against the donor weight and height (Figure 2).

In children, the liver weight in females coincided with that in males. In adults, the male regression line was consistently above the female one. Nonetheless, the gap was non-significant; therefore, we built our model pooling both genders together.

In order to select the variables to build our model, we performed a multivariate analysis in children on the one hand and in adults on the other hand (Supplementary Figure S1).

**Figure 3:**
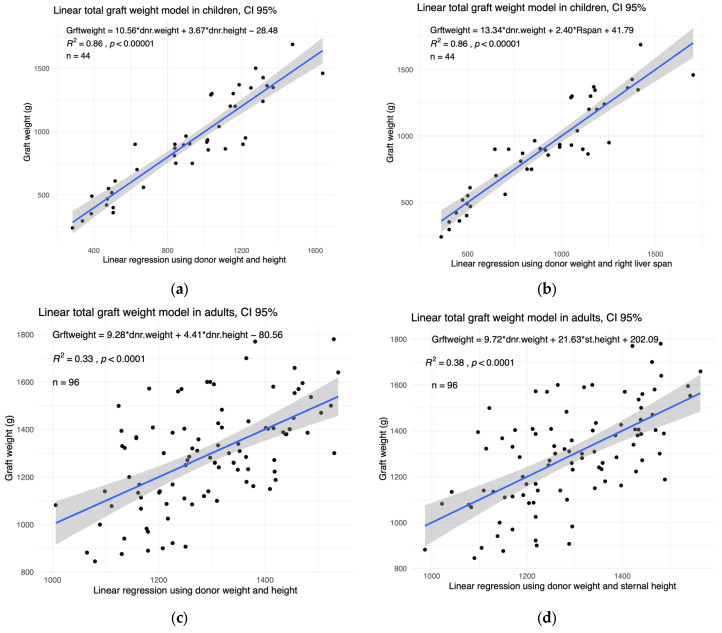
The graft weight can be successfully modelled using linear combinations of the donor weight, height, right liver span, and sternal height. The total graft weight against the optimal linear combinations in (**a**,**b**) children and (**c**,**d**) adults. The *X*-axis represents the linear combination shown in the top left corner of each panel. The R^2^ coefficient of determination and *p*-value of each model are shown in the top left of the corresponding panel. The sample size (*n*) is also shown in the top left. The 95% confidence interval is shown in grey.

We selected the variables which correlate the most with graft weight and removed the variables that correlate strongly between each other in order to avoid redundancy. Two combinations of variables led to the strongest regressions (highest coefficient of determination, R^2^). In children, these were the linear combinations of donor weight and height (AIC = 566) and also donor weight and right liver span (AIC = 553). In Figure 3a,b, we plotted the graft weight as a function of these two optimal combinations.

In adults, the two best combinations were the donor weight and height (AIC = 1247) and the donor weight and sternal height (AIC = 1228) (Figure 3c,d).

Following Urata et al. [9], we tested a logarithmic transformation. Applying the least squares, we found the following: graft weight = (donor weight)^0.449^ × (donor weight)^0.577^ × 2.307. This model yields R = 0.74 and *p* < 10^−4^ (Supplementary Figure S2a); this is a weaker relationship than the linear regressions described above. The same reasoning in children alone yielded a model with R^2^ = 0.85 and *p* < 10^−4^ (Supplementary Figure S2b), and in adults, R^2^ = 0.33 and *p* < 10^−4^ (Supplementary Figure S2c). The logarithmic regression was discarded in favour of the linear regression.

### 3.3. External Validation of Whole Graft Model

**Figure 4:**
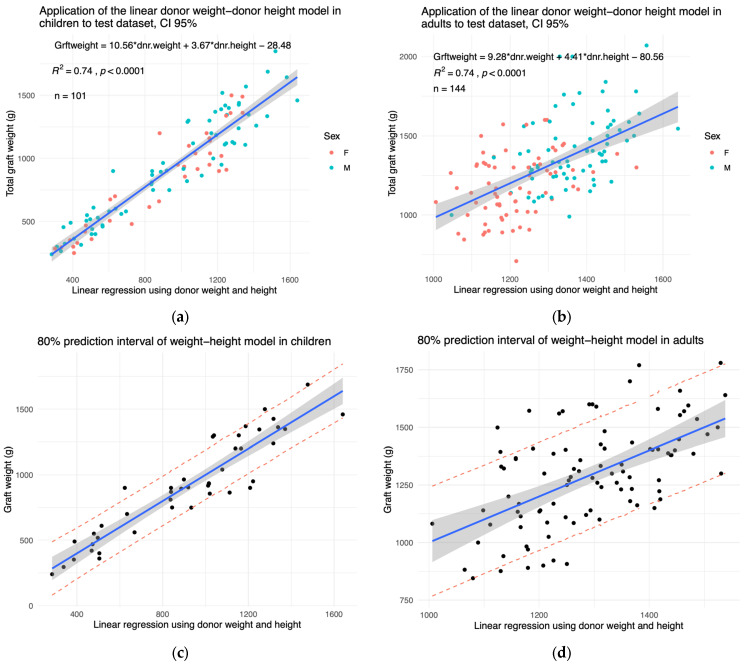
The established models are valid in the test dataset. The graft weights of the test dataset are plotted as a function of the optimal linear regressions established on the test dataset. The *X*-axis represents the linear combination shown in the top left corner of each panel. The tested models were those using (**a**) the donor weight and height in children and (**b**) in adults. An 80 % prediction interval of the (**c**) paediatric model and (**d**) adult model is shown in red dashes. The 95% confidence interval is shown in grey.

The models were tested on a pooled population of patients from another institution and new patients from ours (Table 3 and Table 4). The right liver span was only available for 18 paediatric and 34 adult patients. The sternal height was not available. The graphical representations of the models applied to the test dataset are shown in Figure 4a,b and Supplementary Figure S3a. In children and adults, the tested models are valid on the test dataset. The prediction intervals of our models in children are shown in Figure 4c,d. The confidence interval shows that our model is a satisfactory fit for the training dataset. The 80% prediction interval shows that, should we apply the model to a new child donor from the external validation dataset, we have an 80% certainty that the error margin will not exceed 380 g (Figure 4c, Supplementary Figure S3c). In adults, the prediction interval reached 500 g (Figure 4d, Supplementary Figure S3d). Thus, with 80% certainty, liver weight prediction can over- or underestimate liver weight by a maximum of 190 g in children and 250 g in adults.

### 3.4. User Interface

Having established clinically relevant and mathematically precise models, the next step is to present the model in a user-friendly manner. The interface allowing any surgeon to estimate the weight of a potential donor graft is available online at https://masha-k.shinyapps.io/App-thp/, (accessed on 30 September 2024).

## 4. Discussion

We have successfully established two linear regression models in children and two in adults to predict the total liver weight using exclusively easily accessible biometric donor parameters. The most relevant combinations of variables proved, in children, to be the donor weight and height, as well as weight and right liver span. In adults, these were weight and height and weight and sternal height.

We were able to analyse the impact of seven different donor variables, most of which had high strengths of correlation with the graft weight. We selected those that, together, best predicted our response variable. The models in the paediatric population have a high capacity of predicting the total liver weight (high determination coefficient, R^2^). The adult models have a weaker capacity to predict the graft weight. The coefficient of determination of our adult model was 36%, meaning that only 36% of our data fit the model. The 80% prediction interval was 500 g, meaning that in 80% of the cases, a graft’s real weight is within a 250 g margin of the estimated weight. While such an error can be unacceptable in some clinical situations, our model can be used, if not to accept a graft, then at least to refuse it if the estimated weight is too far from the target weight. Moreover, it can be a useful tool in determining best matches in cases when several are possible. Despite our model’s error margin, these applications could make it a useful tool in the surgeon’s decision-making process.

It may be relevant to continue building our models on a larger training dataset, potentially allowing us to stratify the donors by sex and improve the models’ predictive capacity. Although donors were selected at random, some bias may remain. For instance, the percentage of donors with obesity was under the percentage of overall obesity in our country (17%). Another potential method for improving the models in the future could be to introduce new variables, such as the left liver span. Prospective measuring of the left liver lobes in new donors is currently underway.

By including two variables in each model, we were able to optimise our capacity to predict the response variable. This multivariate approach is both mathematically more precise and more resilient to inter-individual variations. For children, both models are mathematically equivalent. The model using the donor height and weight may be easier to use in clinical practise, as these variables are more frequently measured than the right liver span.

The major goal in liver graft volume prediction is to avoid both small- or big-for-size syndrome [4,5]. In the first case, the transplantations that are considered to be at risk of the phenomenon are those in which the graft liver weighs less than 30% of the recipient’s liver [5]. This cut-off is to be taken into consideration when deciding whether to accept a graft based on the estimated graft weight, especially in the case of whole liver grafts in paediatric patients and when splitting a liver graft between an adult and paediatric team. It is common practise to direct the right part of the liver to an adult team. The paediatric team often receives small lateral segments, as recipients can weigh as little as 5–10 kg. Accepting a whole graft intended for splitting is therefore a difficult decision, which calls for modern and accurate tools. In paediatric liver transplantation, the most important variable to consider is the weight of the left liver lobe. Based on the findings of this study, our next step is to create a model that can predict the weight of the left lobe. This study serves as an initial step in this strategy, helping us to identify and understand the most relevant and accessible variables.

Our models can be readily taken into consideration in a clinical decision; however, it is no more than a tool in the box of the clinician, and other clinically relevant parameters must not be put aside. The models we have established are, to our knowledge, the first mathematically successful attempt at modelling livers using biometric donor variables in the Caucasian population. Previous work by Urata et al. [9] has succeeded somewhat in modelling livers in an Asian population, using both liver harvesting and CT volumetric analysis. Heinemann et al. [10] demonstrated that these formulae tend to underestimate liver weights in the Caucasian population. Donadon et al. [11] compared seven major available models of liver weight in the literature. Several studies [4,9] have used the CT-derived liver volume estimation as a reference. The weights of our donor livers, on the other hand, were directly measured. While CT volumetry is a precise method, the volumetric tool is not available in our national donation allocation system. Therefore, it is not used for volumetric evaluation in our study. Instead, we use a conventional CT scan (slides) to calculate the right hepatic height (routinely reported in the allocation system) and liver–spleen density gradient. In our paediatric team, a steatotic liver (suspected by more than 10 UH spleen–liver density gradient) is systematically discarded in a patient with a high Body Mass Index (BMI > 30), alcoholic habits, or with an age above 40 years. Liver function tests are also used to assess graft acceptance, especially when splitting the liver (transaminases below 80 IU/L are usually required). Our models can be used to inform clinical decisions, but they should be used in conjunction with other relevant parameters, such as the donor’s liver function tests. According to Lim et al. [8], while automated volumetry can take as little as 34 s (0.57 ± 0.06 min/case), semi-automated and manual volumetry can take up to 40 min/case. In our country, with automated volumetry not being standard practise, it may prove more realistic to be able to estimate the graft weight in a matter of seconds. The volumetric tool is important, but our national allocation agency needs to negotiate and purchase it. We intend to fund and carry out this project, but it also needs to be evaluated. One limitation is the small number of paediatric liver transplantations (80/year) in our country compared to the amount of adult liver transplantations (1300/year) [12].

Several models have used estimations of body surface or, alternatively, BMI to predict liver weight. Although body surface estimation and BMI calculation makes use of both the donor weight and height, in our study, we were able to build mathematically stronger models using bivariate linear regressions. Moreover, in paediatric transplantation, using the BMI variable is less relevant than in adults. This is because the BMI limits depend on age in the paediatric population, making them more difficult to interpret before accepting the graft. In the context of paediatric liver transplantation, particularly when splitting the liver and increasing the ischemia time, our team makes a practise of rejecting adult steatotic livers based on CT scan imaging or a BMI above 30 associated with abnormal liver function tests. The models from the literature achieve determination coefficients, R^2^s, of approximately 0.35, meaning that their models are undeniably less successful at predicting liver weight than the models we have established in children.

In conclusion, we have built basic and reliable mathematical models to predict whole liver graft from easily available biometrics variables found in pediatric and adult liver donors. This prediction models are available online at: https://masha-k.shinyapps.io/App-thp/, (accessed on 30 September 2024).

## Figures and Tables

**Table 1:** Statistical parameters of children in the training dataset.

	Number of Values	Number of NA	Min	Max	Median	IQR	Mean
Age (years)	44	0	0	18	12	10	11
Height (cm)	44	0	65	190	151	54	141
Weight (kg)	44	0	7	92	42	36	40
BMI (kg/m^2^)	44	0	11	27	18	4	18
Graft weight (g)	44	0	240	1688	900	654	912
Right liver span (mm)	43	1	89	184	137	49	137
Left liver span (mm)	7	37	48	120	64	25	74
Chest perimeter (cm)	43	1	43	102	70	22	70
Sternal height (cm)	43	1	7	24	16	5.5	16
Abdominal perimeter (cm)	43	1	43	99	63	14	64
Sex M	31	0	NA	NA	NA	NA	NA

BMI: Body Mass Index, NA: not available (missing data), Min: minimal value, Max: maximal value, IQR: inter-quartile ratio.

**Table 2:** Statistical parameters of adults in the training dataset, with two outliers removed.

	Number of Values	Number of NA	Min	Max	Median	IQR	Mean
Age (years)	96	0	19	59	32	23	34
Height (cm)	96	0	158	188	170	10	171
Weight (kg)	96	0	42	89	66	17	67
BMI (kg/m^2^)	96	0	17	30	23	4	23
Graft weight (g)	94	2	845	1780	1300	268	1293
Right liver span (mm)	94	2	100	220	157	36	157
Left liver span (mm)	13	83	45	166	86	33	89
Chest perimeter (cm)	94	2	54	130	91	11	90
Sternal height (cm)	94	2	15	29	20	3	20
Abdominal perimeter (cm)	94	2	66	160	82	15	84
Sex M	45	0	NA	NA	NA	NA	NA

BMI: Body Mass Index, NA: not available (missing data), Min: minimal value, Max: maximal value, IQR: inter-quartile ratio.

**Table 3:** Statistical parameters of children in the test dataset.

	Number of Values	Number of NA	Min	Max	Median	IQR	Mean
Age (years)	101	0	0	18	12	9	11
Height (cm)	101	0	65	190	154	51	141
Weight (kg)	101	0	7	92	44	35	41
BMI (kg/m^2^)	101	0	12	27	19	5	19
Graft weight (g)	101	0	240	1850	910	724	898
Right liver span (mm)	18	83	99	202	160	21	159
Sex M	66	0	NA	NA	NA	NA	NA

BMI: Body Mass Index, NA: not available (missing data), Min: minimal value, Max: maximal value, IQR: inter-quartile ratio.

**Table 4:** Statistical parameters of adults in test dataset.

	Number of Values	Number of NA	Min	Max	Median	IQR	Mean
Age (years)	144	0	19	73	29	21	33
Height (cm)	144	0	150	195	170	13	171
Weight (kg)	144	0	42	95	66	17	67
BMI (kg/m^2^)	144	0	15	29	23	6	22
Graft weight (g)	144	0	709	2070	1300	984	1301
Right liver span (mm)	34	110	90	210	150	32	152
Sex M	73	0	NA	NA	NA	NA	NA

BMI: Body Mass Index, NA: not available (missing data), Min: minimal value, Max: maximal value, IQR: inter-quartile ratio.

## Data Availability

The original contributions presented in the study are included in the article and Supplementary materials, further inquiries can be directed to the corresponding author.

## Supplementary Materials

The following supporting information can be downloaded at: https://www.mdpi.com/article/10.3390/children11101248/s1, Figure S1: Explanatory variables have different strengths of correlation to the response variable (graft weight), and some explanatory variables correlate strongly between each other; Figure S2: Graft weight can be modelled using logarithmic transformations of tested explanatory variables; however, these models are less precise than linear regressions; Figure S3: Graft models using linear regressions of donor weight, right liver span, and sternal height are valid on the test dataset.
