# Supplementary material for: Prediction of Whole Liver Graft Weight Based on Biometric Variables in Paediatric and Adult Liver Donors
Source: Children (Basel). 2024 Oct 16;11(10):1248. doi: 10.3390/children11101248 (PMC11506035; doi:10.3390/children11101248)
Supplement: Supplementary file 1 [file children-11-01248-s001.zip › children-3194241-supplementary.pdf]

children

MDPI

Supplemental Figures

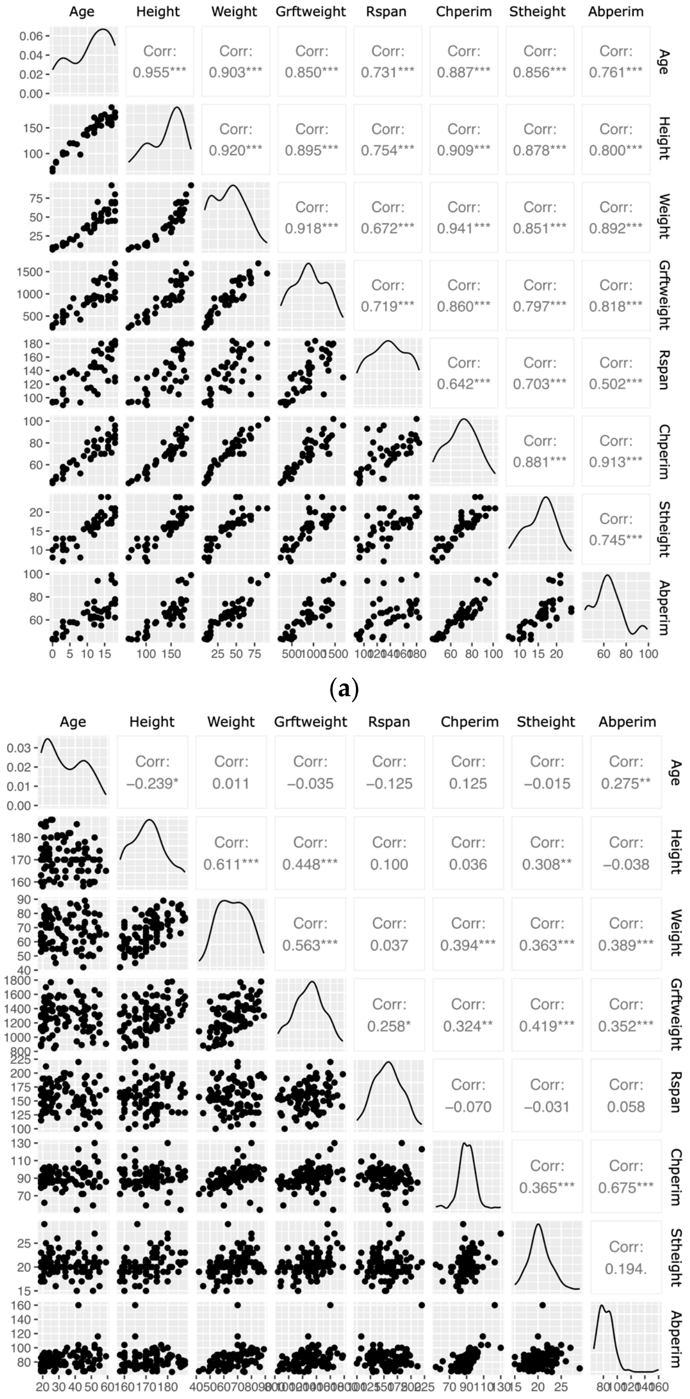

(a)

(b)

Figure S1. **Explanatory variables have different strengths of correlation to the response variable (graft weight), and some explanatory variables correlate strongly between each other.** The parameters of correlations between all variables in (**a**) children and (**b**) adults. Top triangle: Pearson correlation coefficients (R) and p-values (*** $p < 0.001$, ** $p < 0.01$, * $p < 0.05$). Diagonal: density plot for each variable. Bottom triangle: inter-variable correlation plot. Grftweight: graft weight, Rspan: right liver span, Chperim: chest perimeter, Stheight: sternal height, Abperim: abdominal perimeter.

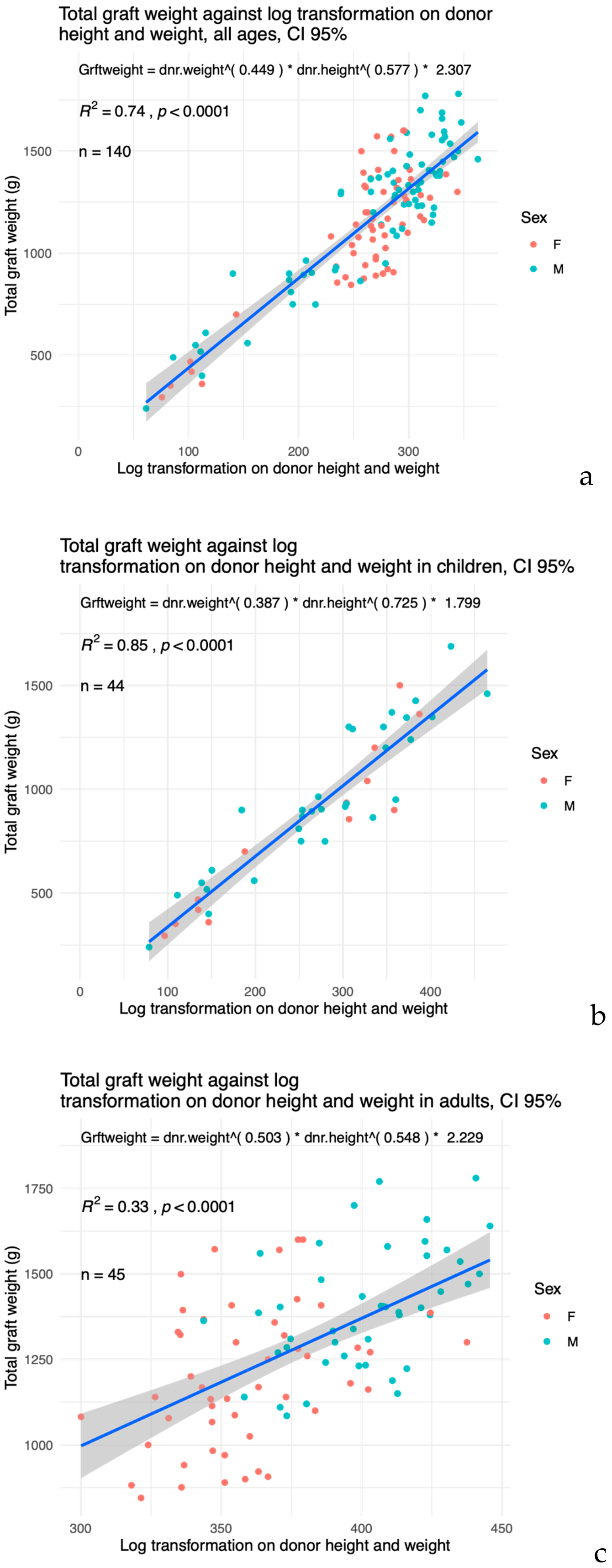

Figure S2. Graft weight can be modelled using logarithmic transformations of tested explanatory variables; however, these models are less precise than linear regressions. The total

graft weight against the optimal logarithmic transformation in (**a**) total population, (**b**) children, (**c**) women, and (**d**) men. The x-axis represents the linear combination shown in the top left corner of each panel. The regression parameters and sample size are shown in the top left. The 95% confidence interval is shown in grey.

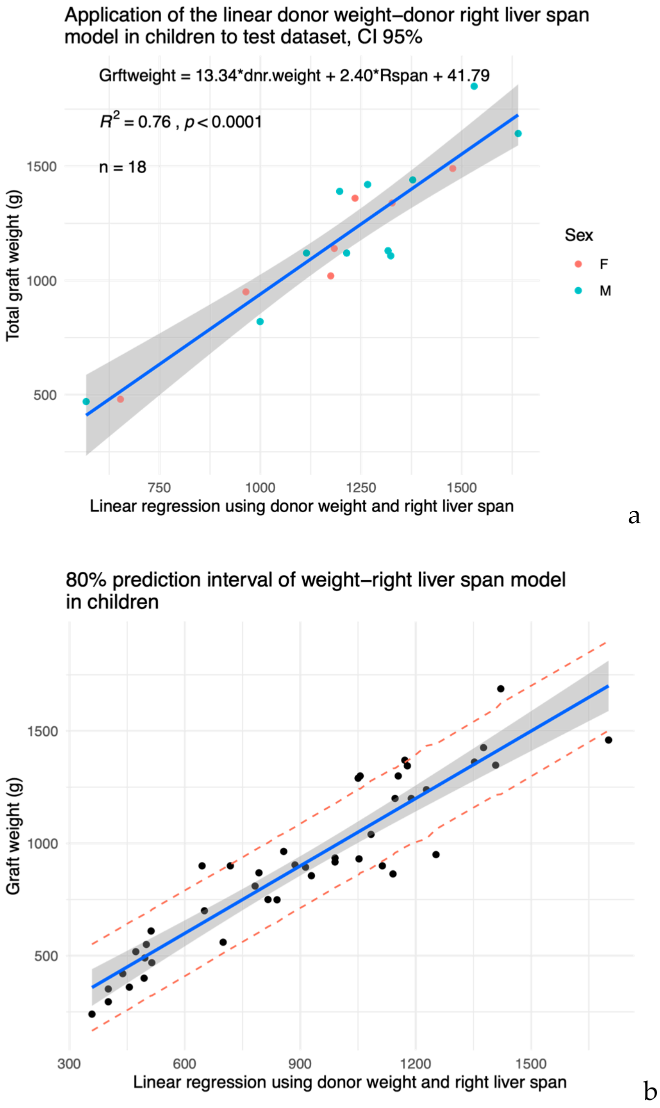

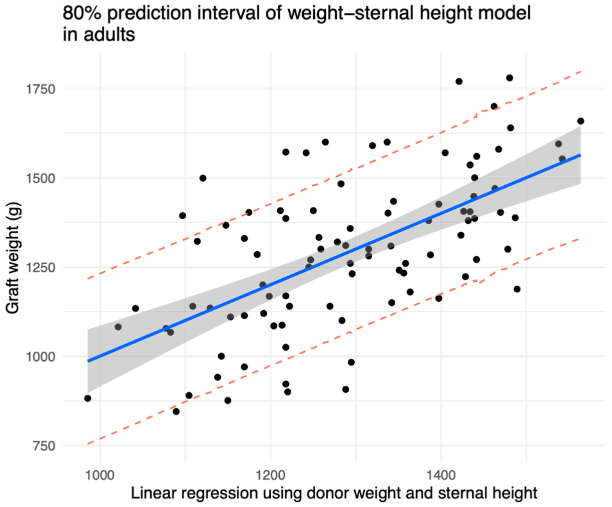

c

Figure S3. Graft models using linear regressions of donor weight, right liver span, and sternal height are valid on the test dataset. The graft weights of the test dataset are plotted as a function of the optimal linear regressions using the donor weight and right liver span or sternal height. The model tested in children (a) is the regression using the donor weight and right liver span. The 80 % prediction intervals of the paediatric model (b) and adult model (c) are shown in red dashes. The 95% confidence interval is shown in grey.
